# Low anterior resection syndrome after rectal cancer surgery: mechanisms, assessment, management, and the potential role of robotic surgery

**DOI:** 10.3389/fonc.2026.1784483

**Published:** 2026-04-01

**Authors:** Chenyu Zhang, Yuzhou Mei, Huiming Wu, Tengfei Wang, Zhenguo Han

**Affiliations:** Department of General Surgery, Third Hospital of Shanxi Medical University, Shanxi Bethune Hospital, Shanxi Academy of Medical Sciences, Tongji Shanxi Hospital, Taiyuan, Shanxi, China

**Keywords:** intervention strategies, low anterior resection syndrome, mechanisms, rectal cancer, robotic surgery

## Abstract

Rectal cancer is one of the most commonly diagnosed malignancies in the digestive system. In recent years, with continuous advances in systemic therapies such as chemotherapy and radiotherapy, as well as the widespread implementation of the multidisciplinary team (MDT) approach, the prognosis for rectal cancer patients has seen substantial improvement. Radical surgical resection, guided by the principle of total mesorectal excision (TME), still forms the foundation of rectal cancer treatment. Recently, the da Vinci surgical robotic system, owing to its high-definition three-dimensional visualization, multi–degree-of-freedom articulated instruments, and stable, flexible camera platform, has been increasingly adopted in radical rectal cancer surgery. With this technological advancement, sphincter preservation has become feasible for a greater proportion of low rectal cancer patients, thereby sphincter-preserving surgery has become feasible for a greater proportion of patients with low rectal cancer. However, the increasing use of sphincter-preserving surgery has been accompanied by a rising incidence of postoperative functional disorders, most notably low anterior resection syndrome (LARS). A combination of symptoms, including increased bowel frequency, urgency, fecal incontinence, and evacuation problems, defines LARS, leading to significant disruptions in bowel function, psychological health, and quality of life. The mechanisms underlying LARS remain incompletely understood. To prevent or alleviate the occurrence of LARS, enhance postoperative bowel function, and improve the quality of life in patients following low anterior resection for rectal cancer, this review summarizes recent advances in rectal cancer–related treatments and provides an overview of the current insights into the mechanisms, assessment, prevention, and management of LARS, aiming to provide evidence-based recommendations for clinical practice.

## 1 Methodology

We conducted a literature search in PubMed, Web of Science, Embase, and Cochrane Library using keywords and MeSH terms including “low anterior resection syndrome,” “rectal cancer,” “robotic surgery,” “laparoscopic surgery,” and “sphincter-preserving surgery.”

The search covered publications from January 2000 to December 2025 and was limited to studies published in English and Chinese. Studies were selected based on relevance to LARS incidence, pathophysiology, prevention, or treatment following rectal cancer surgery. Case reports, conference abstracts without full text, and non-human studies were excluded.

## 2 Background

Colorectal cancer ranks as the third most frequently diagnosed malignancy globally and the second leading cause of cancer-related deaths, with rectal cancer accounting for approximately one-third of all colorectal cancer cases (1). In recent years, the overall mortality rate of colorectal cancer has declined; however, a concerning trend is the exponential increase in the incidence of rectal cancer among individuals younger than 50 years. Surgical treatment remains the cornerstone of multimodal therapy for rectal cancer and represents one of the major challenges in clinical practice (2, 3). Achieving oncological radicality while maximally preserving anal sphincter and genitourinary function has become a central focus of contemporary rectal cancer surgery. Conventional open surgery and laparoscopic approaches are limited by restricted operative space within the narrow pelvis, suboptimal visualization, and limited instrument dexterity, which may result in positive circumferential resection margins, anastomotic leakage, and injury to pelvic autonomic nerves, thereby adversely affecting postoperative bowel and urinary function (4). Pigazzi et al. introduced the use of the da Vinci robotic system in total mesorectal excision (TME) for rectal cancer in 2006, marking the beginning of robot-assisted radical surgery for mid- and low-rectal tumors (5). Thanks to its three-dimensional, high-definition visualization, multi-degree-of-freedom articulated instruments, and effective tremor suppression, the robotic surgical system offers a new technical platform for performing precise, function-preserving, and sphincter-sparing surgery in patients with ultra-low rectal cancer.

Wu et al. (6) conducted a meta-analysis involving 2,239 patients with rectal cancer, revealing that robot-assisted surgery for low rectal cancer is both safe and effective, with a notably reduced rate of postoperative complications compared to traditional surgical approaches. The increasing adoption of robotic surgery has enabled a rising number of patients, especially those with low rectal cancer, to undergo sphincter-preserving procedures. Nevertheless, the rising rate of anus-preserving surgery has been accompanied by an increased prevalence of postoperative functional disorders. These disorders are characterized by a spectrum of bowel dysfunction symptoms, such as frequent bowel movements and challenges with evacuation, fecal incontinence, repetitive painful defecation, and even concomitant urinary incontinence (7). Clinically, this constellation of symptoms resulting from postoperative bowel dysfunction is collectively known as low anterior resection syndrome (LARS) (8). LARS can severely impair bowel function, psychological well-being, and overall quality of life; however, its underlying pathophysiological mechanisms have not yet been fully elucidated. To better prevent or mitigate the occurrence of LARS and to improve postoperative bowel function and quality of life in patients undergoing low anterior resection for rectal cancer, this review provides an overview of recent progress in rectal cancer treatment, with a particular focus on the mechanisms, interventions, and management strategies for LARS, aiming to provide evidence-based insights for clinical practice.

## 3 Robot-assisted radical surgery

### 3.1 Evidence by study type: robot-assisted total mesorectal excision randomized controlled trials

In recent years, accumulating evidence from randomized trials has shown that robot-assisted total mesorectal excision (TME) for rectal cancer is comparable to conventional laparoscopic and open surgery regarding safety and short-term efficacy. The multicenter ROLARR trial compared 237 patients undergoing elective da Vinci robot-assisted rectal cancer surgery with 234 patients treated with conventional laparoscopic surgery, and found no significant differences between the two groups in intraoperative blood loss, conversion to open surgery, or postoperative recovery time (9). The Chinese REAL trial focused on patients with mid- and low-rectal cancer, including a higher proportion of low rectal tumors and patients receiving neoadjuvant chemoradiotherapy. This study demonstrated that, compared with laparoscopic surgery, robotic surgery was associated with significantly less intraoperative blood loss, a lower incidence of postoperative complications, faster postoperative recovery, and lower rates of conversion to open surgery and positive resection margins (10). Regarding long-term oncological outcomes, the REAL trial was the first RCT to designate these as primary endpoints for robotic rectal cancer surgery. Recent results showed that robotic surgery significantly reduced the rate of positive circumferential resection margins (CRM) and yielded higher-quality TME specimens, although whether these advantages translate into long-term survival benefits remains to be determined (10).

#### 3.1.1 Systematic reviews and meta-analyses

Several systematic reviews and meta-analyses have evaluated robot-assisted rectal surgery. These studies generally support the conclusion that robotic surgery reduces intraoperative blood loss and conversion rates compared with conventional laparoscopic surgery. However, evidence regarding other perioperative outcomes, such as time to first flatus and length of hospital stay, remains inconsistent (11–14). Meta-analytic data also suggest potential advantages of robotic surgery in preserving pelvic autonomic nerves, which may contribute to improved early postoperative urinary and sexual function, although long-term functional outcomes require further investigation.

#### 3.1.2 Retrospective and cohort studies

Retrospective cohort studies and single-center analyses provide additional insight into functional and technical outcomes. Studies using the International Prostate Symptom Score (IPSS) and International Index of Erectile Function (IIEF) have reported that robotic surgery may result in better early recovery of urinary and sexual function compared to laparoscopic surgery (15, 16). Subgroup analyses further indicate that robotic surgery may increase sphincter preservation rates, with fewer patients requiring abdominoperineal resection (17). Nonetheless, these findings should be interpreted with caution, as subgroup analyses are often limited by small sample sizes and potential disruption of randomization, which can introduce selection bias.

### 3.2 Robot-assisted surgery, LARS, and long-term bowel function

Recent evidence suggests that the relationship between robot-assisted total mesorectal excision (TME) and postoperative bowel function is best understood in terms of both the severity and the time course of low anterior resection syndrome (LARS), rather than simply its presence or absence. Two meta-analyses indicate that robotic TME is associated with improved anorectal functional recovery, reflected by lower postoperative LARS scores and better long-term anal function than those observed after laparoscopic surgery, open surgery, or transanal TME (18, 19). However, this apparent benefit is more consistent for reducing the overall symptom burden and improving functional scores than for clearly lowering the incidence of major LARS itself (19). A broader systematic review comparing open, laparoscopic, robotic, and transanal TME also highlighted that currently available functional evidence remains limited and heterogeneous, and that clear superiority across approaches has not been consistently demonstrated (20).

Cohort and comparative observational studies further support the possibility of a more favorable recovery trajectory after robotic surgery. In a consecutive robotic rectal cancer cohort, major LARS remained common early after surgery but declined during the first postoperative year, suggesting ongoing bowel functional recovery over time (21). Comparative studies have also suggested better bowel and urinary functional preservation after robotic TME than after transanal or conventional laparoscopic approaches (22). In addition, a propensity score-matched analysis in patients with lower rectal cancer reported that robotic surgery was associated with reduced incidence and severity of LARS during follow-up (23).

Nevertheless, the available evidence should be interpreted cautiously. Extended observational analysis has shown that the minimally invasive approach itself was not independently associated with a lower risk of major LARS, whereas factors such as age, radiotherapy, and low tumor height appeared to be stronger determinants of persistent bowel dysfunction (24). Taken together, current evidence suggests that robotic surgery may improve the severity profile of postoperative bowel dysfunction and may support earlier recovery of bowel function, but whether it meaningfully reduces the long-term incidence of major LARS remains uncertain (19, 24).

## 4 Low anterior resection syndrome

### 4.1 Definition

With the growing understanding of low anterior resection syndrome (LARS), its definition has undergone several revisions and refinements. In 1994, Williamson et al. were the first to highlight alterations in anorectal function following anterior resection of the rectum, defining the postoperative increase in bowel frequency and related symptoms as “anterior resection syndrome” (25). Subsequent studies demonstrated that this syndrome predominantly occurs after low anterior resection for rectal cancer; accordingly, the term was refined to low anterior resection syndrome (LARS).

In 2012, Bryant et al. provided a more systematic description of LARS, defining it as “a clinical syndrome following anterior resection of the rectum, characterized by a range of bowel dysfunctions resulting from alterations in rectal anatomy, pelvic nerve injury, and impaired defecatory reflexes, ultimately leading to deterioration in quality of life” (26). However, this definition was relatively broad and lacked specific diagnostic criteria, limiting its applicability in clinical practice and research.

### 4.2 Pathophysiological mechanisms

In 2020, an international expert consensus standardized and clarified the definition and diagnostic criteria of LARS (27). According to this consensus, a diagnosis of LARS requires a history of anterior resection of the rectum with sphincter preservation, accompanied by at least one characteristic symptom and resulting in at least one adverse consequence. Typical symptoms involve irregular bowel function, altered stool consistency, frequent bowel movements, painful defecation, evacuation difficulties, urgency, fecal incontinence, and fecal leakage. Corresponding adverse consequences include increased dependence on toilet access, excessive concern or dissatisfaction with bowel function, the need for coping strategies, impaired psychological well-being, limitations in social and daily life, reduced interpersonal connections, and broader negative social effects. This consensus offers a unified and actionable approach to the clinical identification, diagnosis, and investigation of LARS, emphasizing not only functional impairment but also its profound impact on patients’ quality of life.

Normal defecatory function relies on the precise coordination of neural regulation, sphincter integrity, and rectal compliance. The pathophysiology of LARS has not been fully clarified and is currently thought to be multifactorial. Among the proposed mechanisms, damage to the anal sphincter and pelvic autonomic nerves during anterior resection and colorectal reconstruction is considered a key contributing factor. Current evidence suggests that LARS is mainly associated with the following mechanisms.

#### 4.2.1 Injury to the internal anal sphincter and perirectal nerves

The anal sphincter complex consists of the internal anal sphincter (IAS) and the external anal sphincter. The IAS, derived from the thickened circular smooth muscle of the intestinal wall, plays a critical role in maintaining resting anal closure and continence. Under normal physiological conditions, sustained contraction of the IAS keeps the anal canal closed, allowing temporary storage of feces in the rectum.

During total mesorectal excision (TME), perirectal neural structures and the IAS are difficult to preserve completely. Bryant et al. reported in 2012 that pelvic surgery—particularly low anterior resection (LAR) for rectal cancer—may damage the IAS and its sympathetic innervation, thereby precipitating LARS. They also demonstrated significant correlations between postoperative IAS pressure, residual rectal length, and fecal incontinence (26).

Animal studies further support this mechanism: denervation of the colon, especially involving the sympathetic nervous system, leads to hypermotility, likely due to unopposed parasympathetic activity, indicating that neural injury can impair IAS function. In addition, mechanical trauma to the sphincter caused by transanal insertion of circular staplers during LAR is another important etiological factor (28). Ho et al. reported that, at 6-month follow-up, patients who underwent stapled anastomosis had significantly lower resting anal pressures and a higher incidence of IAS defects on endoanal ultrasonography compared with those without stapler use. Consequently, injury to the nerves innervating the residual rectum or structural damage to the IAS during surgery predisposes patients to fecal incontinence and impaired voluntary control of defecation (29).

#### 4.2.2 Reduced capacity and compliance of the “neorectum”

Anatomically, the upper rectum has a caliber similar to the sigmoid colon, whereas the distal rectum expands to form the rectal ampulla, which serves as a reservoir and plays a key role in sensing the defecatory urge. After LAR for rectal cancer, the rectal ampulla is usually resected, leaving only a short segment of rectum above the dentate line. The sigmoid or descending colon is then anastomosed to the distal rectum or anal canal, forming a so-called “neorectum”

Because the neorectum has a smaller diameter and reduced capacity, its storage function and compliance are markedly diminished. At the same time, disruption of defecatory reflex pathways further exacerbates bowel dysfunction, leading to increased stool frequency and urgency. Pathological changes such as diverticulitis within the neorectum may further aggravate symptoms. Emmertsen et al. demonstrated that the neorectum exhibits abnormally hyperactive motility patterns after rectal resection, with fecal contents rapidly propelled distally once entering the lumen. Colonic contractile behavior cannot adequately substitute for the reservoir function of the native rectum, resulting in pronounced frequency and urgency of defecation (30).

#### 4.2.3 Preoperative radiotherapy

Radiotherapy plays an essential role in the multidisciplinary management of rectal cancer by inducing tumor cell degeneration and apoptosis and reducing local recurrence. Preoperative radiotherapy can downstage tumors and increase sphincter preservation rates in low rectal cancer; however, it may also adversely affect normal tissues. Radiotherapy is believed to cause neurovascular injury, local tissue fibrosis, and reduced bowel wall compliance, thereby increasing the risk of postoperative LARS.

In addition, radiotherapy can directly impair anal sphincter function, manifested by increased collagen deposition within the IAS, tissue fibrosis, and ultrastructural damage to the external anal sphincter. These changes ultimately reduce resting and squeeze anal pressures, leading to impaired continence and other defecatory disorders (31).

#### 4.2.4 Inflammation and scar formation at the surgical site and anastomosis

In contemporary robotic rectal cancer surgery, linear staplers and circular staplers are widely used for gastrointestinal reconstruction. Staples and sutures around the anastomosis may continuously irritate the neorectum, while scar tissue formed during healing can further compromise bowel function. If anastomotic leakage occurs—regardless of whether reoperation is required—local infection can provoke marked rectal and pelvic inflammation, characterized by hyperemia, edema, increased mucosal secretion, and impaired water absorption.

Direct stimulation of the bowel by inflammatory secretions may enhance intestinal motility, resulting in urgency and increased bowel frequency, thereby worsening LARS and reducing quality of life. Chronic inflammation may also lead to fibrosis, bowel wall stiffness, and reduced compliance, ultimately impairing normal defecatory function (32).

#### 4.2.5 Impaired anal sensory function

Under normal conditions, contact between small amounts of bowel contents and the anal canal mucosa activates sensory receptors that send impulses to the brain, creating the urge to defecate. The cerebral cortex integrates these signals with environmental cues to determine whether defecation should proceed. When the rectum is distended, the anal canal reflexively relaxes and distinguishes between solid, liquid, or gaseous contents, a process referred to as the rectoanal inhibitory reflex.

During LAR for rectal cancer, the anastomosis is often constructed near the dentate line, inevitably disrupting this reflex pathway and diminishing the ability to distinguish stool characteristics. Because anal sensory function is crucial for fine continence control, its impairment may result in fecal leakage during flatus or overt fecal incontinence (33).

#### 4.2.6 Risk factors of LARS

The incidence and severity of low anterior resection syndrome (LARS) are influenced by multiple, interacting factors. These can be broadly categorized as surgical/technical, anatomical, biological, and treatment-related factors, each contributing differently to postoperative bowel dysfunction.

##### 4.2.6.1 Surgical factors

Intraoperative sphincter injury: Accidental damage during dissection reduces continence control.

Anastomotic trauma and fibrosis: Excessive tissue manipulation or tension can increase inflammation and scar formation, impairing neorectal compliance.

Anastomotic leak: Leakage can provoke local inflammation and fibrosis, worsening urgency, stool frequency, and continence.

Diverting stoma and timing of closure: Prolonged diversion may delay neorectal adaptation and early functional recovery.

##### 4.2.6.2 Anatomical factors

Low anastomotic height: Anastomoses very close to the anal verge reduce neorectal reservoir capacity, increasing urgency and incontinence.

Loss of neorectal reservoir: Extensive rectal resection diminishes storage and buffering capacity.

Intersphincteric resection (ISR): Partial removal of the internal sphincter compromises continence.

Baseline anal canal capacity: Smaller anal canal or lower resting tone predisposes to postoperative incontinence.

##### 4.2.6.3 Biological factors

Impaired anal sensory function: Disruption of rectoanal inhibitory reflex reduces the ability to distinguish stool type and consistency, leading to fecal leakage.

Age and comorbidities: Older age, diabetes, or neuropathies reduce recovery potential and exacerbate bowel dysfunction.

##### 4.2.6.4 Treatment-related factors

Neoadjuvant radiotherapy: Can damage autonomic nerves and rectal tissue, decreasing neorectal compliance and increasing urgency and frequency.

Adjuvant chemoradiotherapy: May further impair postoperative bowel function.

### 4.3 Robotic surgery and its limitations in mitigating LARS risk factors

Robotic-assisted surgery has significantly advanced minimally invasive rectal cancer surgery by providing high-definition three-dimensional visualization, articulated instruments, and stable instrument control. These features allow more precise dissection within the confined pelvic space, potentially improving identification and preservation of pelvic autonomic nerves and minimizing inadvertent injury to the internal and external anal sphincters. Consequently, robotic surgery may partially mitigate surgery-related technical risk factors, such as intraoperative sphincter injury, anastomotic trauma, fibrosis, and leak, which are known contributors to early postoperative bowel dysfunction.

However, the impact of robotic surgery on overall LARS incidence and severity is limited. Key limitations include:

#### 4.3.1 Anatomical constraints

Low anastomotic height, loss of neorectal reservoir, and intersphincteric resections are dictated by tumor location and surgical oncologic requirements. Robotic precision cannot increase neorectal capacity or alter essential anatomy.

#### 4.3.2 Biological factors

Baseline anal canal capacity, anal sphincter function, age, and comorbidities remain unaltered by the surgical platform.

#### 4.3.3 Treatment-related factors

Neoadjuvant or adjuvant radiotherapy may damage pelvic nerves and rectal tissue independently of surgical technique.

#### 4.3.4 Evidence limitations

Systematic reviews and retrospective analyses indicate that while robotic surgery may improve short-term functional outcomes compared to open or laparoscopic approaches, the surgical approach alone is not an independent predictor of severe LARS (9, 34–37). Long-term, clinically meaningful improvement in bowel function has yet to be consistently demonstrated.

In summary, robotic surgery can reduce technical intraoperative risks, but the development and severity of LARS remain primarily determined by anatomical, biological, and treatment-related factors. Its role should therefore be interpreted as risk mitigation for select technical factors, rather than a comprehensive solution to postoperative bowel dysfunction.

## 5 Assessment tools for LARS

Currently, more than ten assessment scales and instruments are available to assess bowel function following sphincter-preserving surgery for rectal cancer. Commonly used tools include the Low Anterior Resection Syndrome (LARS) score, Memorial Sloan Kettering Cancer Center Bowel Function Instrument (MSKCC BFI), Wexner fecal incontinence score, Fecal Incontinence Severity Index (FISI), St. Mark’s fecal incontinence score, Bristol Stool Form Scale, Vaizey incontinence score, Fecal Incontinence Quality of Life Scale (FIQL), EORTC QLQ-C30/QLQ-CR29, and Xu Zhong five-item ten-point scoring method (38). These instruments differ in their assessed dimensions, applicable contexts, and specific focus (**Table 1**). Below is a summary of the frequently employed scales for the comprehensive assessment of LARS and bowel incontinence in clinical practice.

**Table 1:** Commonly used instruments for assessing anorectal function in LARS patients.

Name	Type	Items/dimensions	Main contents	Score range	Characteristics
LARS score	Disease-specific (colorectal cancer)	Five items	Incontinence for Flatus; incontinence for liquid stools; Frequency of bowel movements; clustering of stools; urgency	0–42 points 0–20: No LARS; 21–29:Minor LARS;30–42: Major LARS	Concise and easy to administer; suitable for rapid screening and initial clinical evaluation; widely used in clinical practice
MSKCC BFI	Disease-specific (colorectal cancer)	Eighteen items; three dimensions	Frequency-related symptoms; dietary impact; urgency/soilage	18–90 points (higher scores indicate better bowel function)	Comprehensive and detailed assessment of bowel function; suitable for in-depth symptom evaluation; scoring system is complex and time-consuming, which may limit routine clinical use
Xu Zhong method: five-item ten-point scoring system	Disease-specific (colorectal cancer)	Five items	Sensation of defecation; bowel control; sensory function; defecation frequency; defecation time	0–10 points (9–10: excellent; 7–8: good; 5–6: fair; 0–4: poor)	Combines subjective and relatively objective indicators; allows quantitative differentiation of symptoms; provides a comprehensive and reliable evaluation
Wexner score	Symptom-specific (fecal incontinence)	Five items	Flatus incontinence; liquid stool incontinence; solid stool incontinence; use of pads; lifestyle alteration	0–20 points (higher scores indicate more severe fecal incontinence)	One of the earliest scoring systems; simple and easy to use; focuses on fecal incontinence rather than overall LARS; subjectivity and potential overlap among items

### 5.1 LARS score

The LARS score is one of the most widely used disease-specific tools for evaluating bowel dysfunction after low anterior resection and is valued for its simplicity, reliability, and clinical applicability. Initially proposed in 2012, it has since been translated into over 35 languages and validated in multiple international multicenter studies (39).

The Low Anterior Resection Syndrome (LARS) score is one of the most widely used disease-specific instruments for evaluating bowel dysfunction after low anterior resection and is valued for its simplicity and clinical utility. Developed by Emmertsen and Laurberg in 2012 (40), the score comprises five items: incontinence for flatus, incontinence for liquid stools, frequency of bowel movements, clustering of stools, and urgency. Each response category is assigned a weighted score according to its impact on quality of life. The total score ranges from 0 to 42 and is classified as no LARS (0–20), minor LARS (21–29), and major LARS (30–42). Because of its brevity and ease of administration, the LARS score is particularly suitable for rapid screening and initial clinical evaluation; however, as a symptom-based instrument, it may not fully capture all dimensions of bowel dysfunction or individual variability, and is therefore often complemented by other functional or quality-of-life measures.

### 5.2 Memorial sloan kettering cancer center bowel function instrument

The Memorial Sloan Kettering Cancer Center Bowel Function Instrument (MSKCC BFI) is an 18-item multidimensional scale assessing frequency, dietary impact, and defecation experience after rectal surgery (41). With scores ranging from 18 to 90, higher values indicate better bowel function. Owing to its comprehensive structure, it is well suited for longitudinal monitoring, although its complexity may limit routine clinical application.

### 5.3 Xu Zhong five-item ten-point scoring method

The Xu Zhong method assesses bowel function across five domains: defecation sensation, control ability, sensory function, frequency, and duration. Scores range from 0 to 10 and are categorized as excellent, good, fair, or poor, with higher scores indicating better function.

By combining subjective symptoms with relatively objective parameters, it provides a concise and practical functional evaluation in clinical settings (42).

### 5.4 Fecal incontinence questionnaires

The Wexner score is the most widely used instrument for assessing fecal incontinence. It evaluates five domains—flatus, liquid and solid stool incontinence, pad use, and lifestyle alteration—with scores ranging from 0 to 20, where higher scores indicate greater severity (43).

Other scales, such as the Vaizey and Pescatori scores, are also available but are less commonly used (44–46). As these instruments focus specifically on incontinence symptoms, they do not fully capture the overall burden of LARS or its impact on quality of life. Therefore, they are often applied as complementary tools alongside disease-specific or quality-of-life measures.

### 5.5 Clinical application and selection strategy

In routine practice, assessment tools should be selected according to clinical purpose and symptom profile rather than used indiscriminately.

The LARS score is most suitable for rapid screening and initial outpatient evaluation due to its simplicity and disease-specific design. However, it is less appropriate for detailed longitudinal monitoring.

For structured follow-up and research settings, the Memorial Sloan Kettering Cancer Center Bowel Function Instrument (MSKCC BFI) provides a more comprehensive evaluation and is better suited for tracking functional changes over time.

The Xu Zhong method may serve as a concise grading tool in routine settings, while fecal incontinence scales such as the Wexner score are best used as complementary instruments in patients with predominant incontinence symptoms.

A stepwise approach may optimize clinical efficiency: initial screening with the LARS score, followed by targeted evaluation using multidimensional or symptom-specific tools when indicated. This strategy enhances practicality while maintaining comprehensive assessment.

In addition, assessment should ideally be performed at defined time points to facilitate longitudinal comparison. A practical schedule may include a preoperative baseline, reassessment after stoma closure, and postoperative follow-up at approximately 3, 6, and 12 months, with further evaluation tailored to symptom persistence and severity.

## 6 Prevention and treatment of LARS after robot-assisted radical surgery for rectal cancer

### 6.1 Preventive measures

To date, no unified guidelines exist for the prevention and management of LARS. Improving surgical quality, selecting appropriate reconstruction methods, and carefully determining the indications for sphincter-preserving surgery are considered key approaches for LARS prevention.

### 6.2 Bowel reconstruction methods

Reconstruction technique is an important factor influencing postoperative bowel function after sphincter-preserving rectal surgery. Among the commonly used reconstructive options, colonic J-pouch (CJP) anastomosis and side-to-end anastomosis have been widely studied as strategies to reduce the severity of LARS (47).

CJP refers to the creation of a colonic J-shaped reservoir using the proximal colon after rectal resection, with the aim of improving neorectal capacity and buffering function. It was introduced to compensate for the loss of rectal reservoir function following low anterior resection. Compared with straight coloanal or colorectal anastomosis, CJP may improve postoperative bowel function, particularly in the early postoperative period, by reducing stool frequency, urgency, and fragmentation (48). However, the functional advantage may diminish over time in some patients. In clinical practice, CJP may be particularly useful in patients undergoing very low rectal resection when sufficient colon length and favorable pelvic anatomy allow pouch construction. Its limitations include technical complexity, the need for adequate colonic length and mobility, and reduced feasibility in patients with a narrow pelvis, obesity, bulky mesocolon, or tension at the anastomotic site.

Side-to-end anastomosis, first introduced by Baker in 1950, is a technically simpler alternative that also provides a degree of reservoir effect (49). It is often considered when construction of a colonic J-pouch is difficult or impractical, such as in patients with a narrow pelvis or limited colonic reach. Available evidence suggests that side-to-end anastomosis may provide bowel functional outcomes comparable to CJP in many cases, while offering advantages such as easier construction and shorter operative time. However, the choice between CJP and side-to-end anastomosis should be individualized according to pelvic anatomy, tumor location, colonic mobility, anastomotic tension, and surgeon experience. Overall, both techniques may be preferable to straight anastomosis in selected patients, but further high-quality comparative studies are still needed to clarify their long-term functional differences.

### 6.3 Pelvic floor rehabilitation

Pelvic floor rehabilitation, including pelvic floor muscle training (PFMT), biofeedback training (BFT), and rectal balloon training (RBT), is currently one of the most important non-invasive interventions for improving anorectal function, treating fecal incontinence, and alleviating LARS, with overall effectiveness ranging from 50%–80% (50).

PFMT involves guiding patients to actively contract and relax pelvic floor and anal sphincter muscles to enhance support and muscle strength. It is simple, non-invasive, and has high patient adherence (41). Studies indicate that initiating PFMT during hospitalization and continuing for six months post-discharge is an effective intervention for LARS (51). Asnong et al. (52) reported that starting PFMT one month after rectal cancer surgery or protective stoma reversal improved LARS rates by 18.76% at 4 months and 26.5% at 6 months compared with controls, with statistically significant differences; however, at 12 months, the difference decreased to 5.1% and was no longer significant. These findings suggest PFMT is more suitable as an early postoperative intervention. Since PFMT relies on patient self-training, its effectiveness depends heavily on patient motivation, self-discipline, and adherence (53). Mobile health and remote guidance have recently been explored to improve adherence and long-term outcomes in LARS management.

BFT is a non-invasive, non-pharmacological approach that provides real-time visual feedback of pelvic floor muscle contraction and relaxation via anorectal manometry or EMG, helping patients perform exercises correctly and effectively (50). RBT trains rectal sensation, compliance, and storage capacity by inflating a balloon to simulate the urge to defecate, thereby improving symptoms of frequency and urgency (50). Studies indicate that combining PFMT, BFT, and RBT achieves better functional improvement than single-modality interventions (54). Wu et al. (55) conducted a 16-month follow-up of 109 sphincter-preserving rectal cancer patients, showing that the combined BFT+PFMT group had a LARS incidence of 22.86%, significantly lower than the PFMT-only group (47.22%), supporting the superiority of multimodal rehabilitation. However, combined therapy often requires regular hospital visits, which may be limited by medical resources, patient economics, and time. Individualized, feasible rehabilitation plans are therefore recommended.

### 6.4 Pelvic floor peritoneum reconstruction

The pelvic floor is a structurally integrated system, and its integrity is crucial for pelvic organ function. Bones, muscles, organs, and ligaments form an interdependent structure; damage to any component may impair pelvic function. Pelvic floor peritoneum reconstruction is widely used in laparoscopic abdominoperineal resection but is less common in sphincter-preserving low rectal cancer surgery. Ji et al. (56) reported that pelvic floor peritoneum reconstruction significantly improved postoperative anal function, with Wexner scores of 3.13 ± 2.79 vs. 4.71 ± 3.45 (P = 0.003) and LARS scores of 21.77 ± 8.62 vs. 25.14 ± 8.78 (P = 0.015). Reconstruction restores normal pelvic anatomy, limits lateral motion of the neo-rectum, and isolates the abdominal cavity from the presacral space, preventing small bowel descent from compressing the neo-rectum and improving bowel function. This approach may reduce the risk of postoperative LARS and provides functional protection for sphincter-preserving surgery.

### 6.5 Greater omentum transplantation

The greater omentum has unique biological properties for bowel reconstruction. As autologous tissue, it preserves its volume and biological characteristics when transplanted into the presacral space, creating a mesenteric fat-wrapped structure around the neo-rectum that mimics the original rectal mesentery and prevents adhesions, maintaining compliance. Qin et al. (57) reported that omental flap transplantation into the presacral space significantly improved postoperative anal function without increasing operative time or technical difficulty. However, clinical cases remain limited, and larger multicenter RCTs are needed to validate efficacy and safety.

### 6.6 Perioperative management improvements

Patients often receive extensive preoperative information about cancer and survival and may expect a return to normal life after surgery, yet they may underestimate the burden of postoperative bowel dysfunction. Health-related quality of life (HRQoL) is an important postoperative outcome, and surgeons may not fully appreciate the impact of bowel dysfunction. Battersby et al. (58)played a pivotal role in the development and external validation of the POLAR score. Their study demonstrated the effectiveness of the POLAR score in predicting bowel dysfunction after rectal cancer surgery, showing that the POLAR score accurately identifies patients at higher risk of developing severe LARS. This predictive power is crucial for developing preventive measures and selecting appropriate treatment options.

Wu et al. (27)examined the impact of postoperative functional management on the recovery of anal function in patients undergoing robotic total intersphincterectomy. Their findings support the use of the POLAR score to identify patients who require more intensive rehabilitation interventions to manage bowel disorders.

The relevance of the POLAR score in the robotic surgical setting has been further verified, including the comparison of robotic-assisted and traditional laparoscopic surgery. Keane et al. (33)found that the precision of robotic surgery helps reduce nerve damage and enhance sphincter preservation. However, the POLAR score remains crucial for assessing the full spectrum of postoperative bowel dysfunction, emphasizing the importance of incorporating it into comprehensive postoperative evaluations, even for patients undergoing minimally invasive procedures.

Overall, the POLAR score provides a useful framework for predicting, evaluating, and guiding the management of bowel dysfunction after rectal cancer surgery.

### 6.7 Conceptual framework of LARS: integration of mechanisms, prevention, and treatment

**Figure 1:**
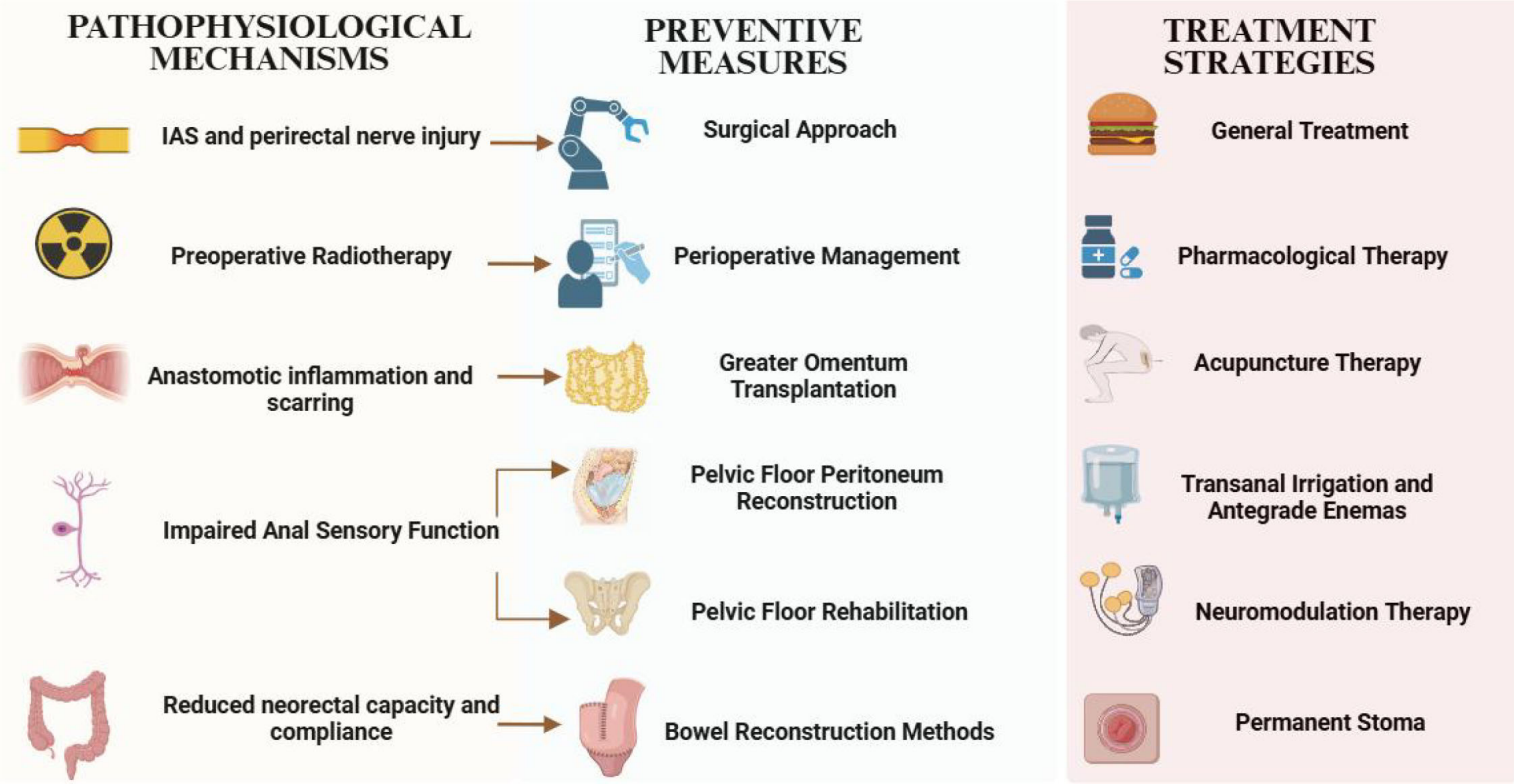
Conceptual framework of low anterior resection syndrome (LARS): integration of pathophysiological mechanisms, preventive measures, and treatment strategies.

To provide a comprehensive visual synthesis of the multifactorial nature of LARS and the interrelationships between its underlying mechanisms, preventive strategies, and therapeutic approaches, **Figure 1** integrates these key elements into a conceptual framework. In this figure, arrows indicate the corresponding relationships between specific pathophysiological mechanisms and their targeted preventive measures.

## 7 Treatment of LARS

Currently, high-quality evidence-based guidelines for managing LARS are lacking, and no unified clinical consensus exists. Existing strategies are mostly extrapolated from management approaches for functional constipation and fecal incontinence. Clinical observations indicate that most LARS symptoms gradually improve within 1–2 years postoperatively, but a subset of patients with severe LARS experience persistent symptoms for several years, significantly impacting quality of life. Based on symptom severity and impact on daily life, a “stepwise” comprehensive treatment strategy has been proposed, progressing from non-invasive measures to more invasive interventions for individualized management. The BOREAL study proposed a stepwise escalation treatment regimen and conducted corresponding clinical practices, achieving favorable outcomes and is expected to become a standardized protocol in the future (59). **Table 2** summarizes this staged treatment approach.

**Table 2:** The BOREAL stepped-care bowel rehabilitation program.

Treatment step	Assessment Time Point	Target Population	Intervention
Step 0	Hospital discharge	All patients at hospital discharge	Routine initiation of anti-diarrheal agents Low-residue diet guidance
Step 1	Postoperative day 30	Patients with major LARS at 30-day assessment	Addition of bulking agents or enemas for patients with major LARS
Step 2	Postoperative month 3	Patients with persistent major LARS at 3-month assessment	Pelvic floor physical therapy Biofeedback training Transanal irrigation
Step 3	Postoperative month 6	Patients with persistent major LARS at 6-month assessment	Sacral nerve modulation
Step 4	Postoperative month 9	Patients with persistent major LARS at 9-month assessment	Percutaneous endoscopic caecostomy Antegrade enema
Step 5	Postoperative month 12	Patients with persistent LARS symptoms beyond 12 months	Definitive colostomy

### 7.1 General treatment

General treatment forms the foundation of LARS management and primarily includes self-behavioral interventions and dietary modifications. Self-management is simple and safe, including regular physical activity, adequate hydration, establishing regular bowel habits, proper defecation posture, and maintaining a healthy lifestyle. Dietary adjustments are also critical, avoiding foods that exacerbate diarrhea or soften stool, such as caffeinated drinks, citrus fruits, spicy foods, and alcohol. Studies suggest that carboxymethyl cellulose may increase fecal incontinence frequency and should be used cautiously. Staller et al. conducted a prospective study on over 60,000 elderly women, demonstrating that a long-term intake of dietary fiber significantly reduced the risk of fecal incontinence (60). Liu et al. also found that fat and meat intake within 6 months postoperatively was significantly associated with LARS improvement, and alcohol cessation was an independent factor for symptom relief (61).

### 7.2 Pharmacological therapy

Pharmacological treatment is an important intervention for mild-to-moderate LARS and is often based on previous experience with diarrhea and fecal incontinence. Loperamide, a μ-opioid receptor agonist, is widely used to reduce bowel frequency and improve incontinence by inhibiting intestinal motility and secretion. However, long-term use may cause refractory constipation or toxic colitis, so it is generally used short-term or intermittently.

Recently, 5-hydroxytryptamine 3 (5-HT_3_) receptor antagonists have been used to alleviate severe LARS symptoms (62). Serotonin plays a key role in diarrhea-predominant irritable bowel syndrome (IBS-D), with elevated serum 5-HT levels and enhanced intestinal motility. Atkinson et al. (63) demonstrated that 5-HT_3_ antagonists delayed defecation and improved stool consistency and urgency. Ramosetron, a highly selective 5-HT_3_ antagonist with good affinity and tolerability, has shown efficacy in IBS-D (64). Itagaki et al. (65) treated 25 severe LARS patients with ramosetron for one month, resulting in significant reductions in incontinence scores and daily bowel movements, suggesting potential value despite limited sample size. Probiotics may improve diarrhea by modulating gut microbiota, but evidence for LARS remains inconclusive. Other adjunctive drugs include anticholinergics such as atropine and scopolamine.

### 7.3 Transanal irrigation and antegrade enemas

When general and pharmacological therapies are insufficient, transanal irrigation can serve as an effective second-line treatment. By regularly emptying the bowel, it simulates normal defecation patterns, promoting rectal evacuation and reducing involuntary stool passage. With proper training, patient adherence and satisfaction are generally high. Early postoperative “preventive transanal irrigation” (~3 months) has shown better LARS improvement compared to supportive care or drug therapy, without serious adverse events. Antegrade enemas, delivered via percutaneous endoscopic colostomy, appendicostomy, or ileostomy, offer an alternative method. Didailler et al. (66) reported significant LARS score improvement in patients receiving antegrade enemas, with ~88% avoiding permanent colostomy, indicating potential as a treatment for refractory LARS. However, these methods are invasive and carry risks such as bowel perforation, requiring careful risk-benefit assessment and patient counseling.

Although transanal irrigation has shown promise in improving LARS symptoms, the existing evidence is limited by small sample trials, short-term follow-up, and high dropout rates, lacking robust randomized data to confirm it as the most effective treatment. Therefore, Klimovskij et al. (67)initiated a multicenter randomized clinical trial to assess whether transanal irrigation can improve intestinal function and quality of life in patients after low anterior resection.

### 7.4 Neuromodulation therapy

Neuromodulation can be non-invasive or invasive. Non-invasive approaches include percutaneous tibial nerve stimulation (PTNS), while invasive methods primarily involve sacral nerve stimulation (SNS).

SNS is a minimally invasive surgical intervention performed in two stages: a test phase to identify responders, followed by permanent implantation if symptoms improve. The device is usually implanted subcutaneously in the buttock, with stimulation parameters adjustable via an external controller. Because LARS symptoms fluctuate, frequent adjustments and long-term follow-up are essential. Dulskas et al. (2015) proposed PTNS as a lower-risk, simpler, and less costly alternative, showing superior short-term efficacy compared to medications, with comparable results at one year (68).

### 7.5 Permanent stoma

For patients with severe LARS unresponsive to multiple therapies, particularly those with symptoms lasting over 2 years and significantly affecting quality of life, permanent stoma may be considered. It can relieve symptoms and improve quality of life, with some patients requesting the procedure. However, robust evidence confirming long-term benefit is lacking. Preoperative counseling must discuss potential benefits (relief of urgency, incontinence, anal pain) and possible complications (parastomal hernia, prolapse, skin irritation, leakage) to ensure informed decision-making.

### 7.6 Acupuncture therapy

According to Traditional Chinese Medicine (TCM), patients with rectal cancer may have preexisting spleen and stomach deficiencies, which can be further aggravated by surgery, potentially contributing to increased stool frequency, urgency, and fecal incontinence. Acupuncture aims to regulate bowel function by targeting acupoints associated with gastrointestinal and pelvic floor control. For example, Dulskas et al. (69) applied acupuncture to points such as Zusanli and Yintang in LARS patients and reported modest symptom improvement.

Acupuncture represents a non-pharmacological, minimally invasive approach that may be considered for mild-to-moderate LARS or patients who cannot tolerate conventional therapies. However, the available evidence is limited by small sample sizes and methodological heterogeneity. Therefore, acupuncture should be regarded as an exploratory or adjunctive option, and further well-designed randomized controlled trials are needed to establish efficacy, safety, and optimal treatment protocols.

### 7.7 Summary of therapeutic interventions for LARS

**Table 3** provides a structured overview of therapeutic interventions for LARS, including clinical indications, timing, expected benefits, limitations, adverse effects, and evidence quality, to aid clinical decision-making.

**Table 3:** Summary of therapeutic interventions for LARS.

Treatment	Clinical indication	Duration	Expected benefit	Limitations	Adverse effects	Evidence level
Pelvic Floor Muscle Training (PFMT)	Occasional leakage or mild urgency, daily life mostly manageable	Start 4–6 weeks post-op; continue 6–12 weeks	Strengthen sphincter, reduce urgency and incontinence	Requires adherence and guidance; less effective in severe injury	Minimal	Moderate–High
Biofeedback Training	Persistent leakage or urgency	Concurrent with PFMT, 6–12 weeks	Improves neuromuscular coordination, reduces symptoms	Time-intensive, resource-dependent	Minimal	Moderate
Transanal Irrigation	Frequent urgency, incontinence, refractory symptoms	Start ~3 months post-op, individualized	Improves bowel emptying, reduces urgency and leakage	Labor-intensive; learning curve	Mild abdominal discomfort, mucosal irritation	Low–Moderate
Pharmacological Therapy	Diarrhea-predominant or urgent symptoms	As needed, dose titrated	Reduces stool frequency, improves continence	Symptomatic only; does not address pathophysiology	Constipation, bloating	Moderate
Neuromodulation	Persistent severe incontinence or urgency	Test phase + implantation; several weeks to months	Improves sphincter function and continence	Invasive; specialized center; variable efficacy	Pain, infection, device issues	Low–Moderate
Permanent Colostomy	Severe, refractory symptoms	Consider after failure of other interventions	Complete symptom resolution, improved QoL	Irreversible; psychological impact	Surgical or stoma-related complications	High (for symptom resolution)
Acupuncture	Intermittent urgency, increased bowel frequency, or adjunctive use when standard therapies are insufficient	Exploratory, variable	Potential modest improvement in urgency and frequency	Small sample size, heterogeneous protocols	Minimal	Low

## 8 Discussion

The robotic surgical system was developed to address several technical limitations of conventional laparoscopic rectal surgery, particularly in visualization, instrument flexibility, and precision within the narrow pelvic space. As reflected in the current literature, robot-assisted rectal cancer surgery appears to be safe and feasible, with short-term perioperative outcomes generally comparable to or, in selected aspects, better than those of laparoscopic surgery. The platform may offer technical advantages in complex pelvic dissection, autonomic nerve identification, and sphincter preservation. However, although these technical benefits are clinically relevant, they should not be assumed to translate directly into consistent long-term functional improvement. In particular, greater sphincter preservation or improved nerve visualization does not necessarily result in better postoperative bowel function, because the development of LARS is multifactorial and extends beyond surgical technique alone.

LARS remains one of the most important determinants of long-term quality of life after sphincter-preserving rectal cancer surgery. This review highlights that postoperative bowel dysfunction is influenced by a complex interplay of anatomical, neural, inflammatory, and treatment-related factors, including low anastomotic height, loss of reservoir function, radiotherapy, anastomotic complications, and pelvic autonomic nerve injury. Within this framework, robotic surgery may mitigate some surgery-related technical risks, particularly those related to pelvic dissection and nerve preservation, but it cannot fully overcome anatomical constraints or treatment-related injury. Current evidence suggests that robotic surgery may improve certain early functional outcomes and may reduce the overall severity of bowel dysfunction in selected settings, yet its effect on the long-term incidence of major LARS remains uncertain.

Several important limitations in the current body of evidence should be acknowledged more explicitly. First, substantial heterogeneity persists in the definition and reporting of LARS across studies, despite the publication of international consensus criteria. Second, functional assessment time points vary widely, ranging from early postoperative months to several years after surgery, making direct comparison difficult and potentially obscuring the natural evolution of bowel recovery. Third, differences in diverting stoma use, timing of stoma closure, neoadjuvant treatment, reconstruction technique, and perioperative management may all confound postoperative functional outcomes but are inconsistently controlled across studies. Fourth, many available data on robotic functional outcomes are derived from retrospective cohorts, subgroup analyses, or heterogeneous meta-analyses rather than high-quality prospective trials specifically designed with bowel function as a primary endpoint. As a result, the currently available evidence is insufficient to draw firm conclusions regarding the true long-term functional superiority of robotic surgery.

These limitations also define the major priorities for future research. High-quality prospective and randomized studies are needed to evaluate robotic surgery using standardized functional endpoints, including validated LARS-related measures, uniform follow-up intervals, and clinically meaningful long-term assessment beyond the first postoperative year. Future studies should also clarify the long-term functional impact of robotic surgery on bowel, urinary, and sexual function separately, rather than treating postoperative recovery as a single composite outcome. In addition, more evidence is needed to determine the optimal timing, intensity, and combination of rehabilitation strategies, including pelvic floor muscle training, biofeedback, rectal balloon training, and transanal irrigation, especially in patients at high risk of persistent LARS. For more invasive interventions such as neuromodulation or permanent stoma, better patient selection criteria are also required, with attention to symptom severity, duration, quality-of-life impairment, treatment responsiveness, and patient preference.

From a clinical perspective, the management of LARS after rectal cancer surgery should remain individualized and multidisciplinary. Robotic surgery may represent one component of a broader strategy to reduce postoperative dysfunction, but it should not be regarded as a comprehensive solution. Careful patient counseling, risk stratification, standardized functional follow-up, and stepwise rehabilitation remain essential. As robotic technology and postoperative management continue to evolve, integrating surgical precision with patient-centered functional assessment will be crucial for improving both oncologic and quality-of-life outcomes in rectal cancer survivors.

## 9 Limitations

This review has several limitations. First, as a narrative review, it does not follow a predefined systematic review protocol, which may introduce selection bias in study inclusion and interpretation. Second, the available literature on LARS after robotic rectal cancer surgery remains heterogeneous in study design, patient populations, operative techniques, reconstruction methods, neoadjuvant treatment exposure, and follow-up duration, which limits direct comparison across studies. Third, despite growing international consensus, the definition and reporting of LARS are not fully uniform, and the timing of functional assessment varies considerably among studies. Fourth, important factors that may influence postoperative bowel function, such as diverting stoma use and closure timing, perioperative management, and rehabilitation strategies, are inconsistently reported and may confound outcomes. Finally, high-quality evidence specifically addressing long-term robotic-related functional outcomes remains limited, as many currently available data are derived from retrospective analyses or secondary functional endpoints rather than prospective trials primarily designed to evaluate bowel dysfunction.

## 10 Conclusion

Low anterior resection syndrome remains a major long-term challenge after sphincter-preserving surgery for rectal cancer and has a substantial impact on bowel function and quality of life. As discussed in this review, LARS is a multifactorial condition resulting from anatomical changes, impaired neorectal function, sphincter injury, pelvic autonomic nerve damage, radiotherapy, and anastomotic complications. Although robot-assisted surgery offers technical advantages in visualization and pelvic dissection, current evidence does not yet establish a clear long-term functional benefit in preventing or managing major LARS. Robotic surgery may contribute to functional preservation in selected settings, but it should be regarded as one component of a broader multidisciplinary strategy rather than a definitive solution. Further prospective studies with standardized definitions, consistent functional assessment, and long-term follow-up are needed to clarify its true clinical value.
